# Assessing the investment risk: An empirical analysis of Altman’s Z-score model

**DOI:** 10.1371/journal.pone.0354297

**Published:** 2026-07-30

**Authors:** Kazi Afaq Ahmed, Ambreen Fatima, Fouzia Sohail, Khalid Jamal

**Affiliations:** 1 Institute of Business Management, Karachi, Sindh, Pakistan; 2 Applied Economics Research Centre, University of Karachi, Karachi, Sindh, Pakistan; 3 Department of Computer Science, University of Karachi, Karachi, Sindh, Pakistan; Yamanashi Gakuin University: Yamanashi Gakuin Daigaku, JAPAN

## Abstract

Investors can play a significant role in reviving the economy and addressing the national agenda of poverty elimination through job creation. Though foreign and local investment are the backbone of any economy, especially in developing economies like Pakistan, investors face the challenge of assessing investment risk to protect the capital employed and achieve an appropriate return on investment. The aim of this research is to assess investment risk faced by firms in Pakistan using the most suitable approach, and then to develop an economic model for a comprehensive assessment of the factors influencing investors’ risk, focusing on firm-specific characteristics, economic conditions, and political stability. The introduction of the Investment Risk Index based on Altman Z-scores provides a structured diagnostic approach to assessing investment risk in Pakistan. Earlier studies have mainly used financial ratios to explain risk. The estimation results using an analytical approach (the Investment Risk Framework) confirm that firm-specific characteristics and economic, political, and financial stability indicators significantly impact investment risk. The findings, based on 443 firms over the period 2014–2023, reveal that the proportion of firms in the distress zone increased from 4.8% to 8.5% (a doubling) during the study period. Retained earnings relative to total assets emerged as a strongly correlated factor with investment risk, while the market value of equity relative to total liabilities played a significant role in keeping firms (around 88% of the sample) in the safe zone. For the determinants, the GMM results indicate that past values of investment risk strongly predict future risk. A 1% increase in past risk is associated with a 0.395% higher future risk. Additionally, political stability, GDP growth, inflation, current account balance, broad money, dividend premium paid, and growth opportunities are found to have a significant impact on the investment risk. Furthermore, internal factors such as fixed asset accumulation, firm size, and tax expenses also influence risk, with larger firms facing higher risk. This study underscores the importance of understanding investment risk factors for informed decision-making. The findings offer valuable insights for investors and policymakers, with potential applicability to other developing economies.

## 1. Introduction

Investors’ confidence in an economy, crucial for job creation, depends heavily on a stable business environment and low investment risk [1]. Assessing this risk requires considering multiple dimensions, from macroeconomic factors to firm-specific characteristics. The challenge is to assess investment risk to protect capital employed and get an appropriate return on investment. Currently, various reports and datasets are available, such as “The Doing Business Report (World Bank), IRCG (International Country Risk Guide Index – Harvard/MIT dataset), Global Risk Indicators, Report of Rating agencies and financial institutions, etc., to provide some guidance to investors about the investment risk. However, predicting investment risk by employing a diagnostic tool such as the Z-score and integrating it into a comprehensive analytical framework that incorporates firm-specific characteristics, macroeconomic conditions, and political instability remains an understudied area of research in Pakistan.

From an analytical perspective, international studies have explored various dimensions of investment risk, capital structure, financial distress, and economic fluctuations, providing valuable insights into the interplay between internal and external factors the affect investment decisions. For example, in the case of Poland, [2] examines the impact of macroeconomic fluctuations on investment decisions and highlights a strong long-term correlation between corporate investments and macroeconomic indicators, with GDP and public spending being particularly significant. Complementing this macroeconomic perspective, [3] examines the macroeconomic and firm-specific determinants of capital structure in emerging markets, focusing on listed firms in Ghana, Kenya, Nigeria, and South Africa. The findings reveal that firm-specific factors, such as growth opportunities and the cost of debt, significantly influence capital structure, while macroeconomic factors, such as inflation and foreign direct investment, also play a critical role. However, the determinants of leverage vary across countries, underscoring the need for tailored financial regulations and firm-level strategies to optimise capital structure decisions in emerging markets. In a similar vein, [4] analyse firm growth by integrating firm-level, industry-specific, and macroeconomic determinants using data from European firms. The study finds that firm-level factors, such as productivity, intangible assets, and financial structure, have the most decisive influence on growth. At the macroeconomic level, factors like inward FDI, natural resources, and infrastructure positively impact firm expansion, while unemployment and regulatory burdens hinder growth. The research highlights the significance of institutional determinants, including political stability and business environment quality, in shaping firm growth dynamics. The study by [4] contributes to understanding the multi-dimensional drivers of firm expansion, emphasising the interplay between internal capabilities and external economic conditions.

Further, the assessment of investment risk using a diagnostic approach, such as a Z-score-based model, has often remained in focus. Specifically, studies have emphasised the applicability of the Altman Z-score model for assessing bankruptcy and financial distress risk. For example, [5] using the firm-level data, examines the impact of corporate debt, firm size, and financial fragility on financial distress by employing the Altman Z-score. The findings suggest that large firms exhibit higher financial vulnerability despite their systemic importance due to greater exposure to leverage and exchange rate fluctuations. The study also highlights that corporate financial distress is influenced by macroeconomic factors such as currency depreciation, GDP growth, and financial globalisation.

Further, emphasising the applicability of the Altman Z-score, [6] applied the Altman Z-score model to assess bankruptcy risk among multidisciplinary enterprises in Vietnam using financial data from 180 firms. The study examines both financial and non-financial determinants of corporate distress. The results indicate that financial factors, such as earnings before tax, net profit-to-equity ratio, EBIT-to-total assets ratio, and equity-to-total debt ratio, significantly impact bankruptcy risk. Additionally, non-financial variables, including business type, firm size, and managerial education, also influence financial stability. The study highlights the utility of the Z-score model for predicting early bankruptcy and provides policymakers and financial institutions with insights on supporting business sustainability.

The studies discussed above provide a comprehensive view of diagnostic and analytical approaches to assessing investment risk models by integrating firm-level financial indicators with macroeconomic conditions. The insights drawn from these works contribute to a more comprehensive understanding of investment risk. This can guide policymakers, financial institutions, and investors in developing robust risk assessment frameworks tailored to a dynamic economic environment, a topic that has not been comprehensively discussed in the case of Pakistan.

The existing literature on investment risk in Pakistan primarily focuses on sector-specific studies. For instance, [7] employed Altman’s Z-score to measure investment risk in a sample of 21 non-financial firms and found that liquidity, efficiency, and solvency ratios are positively associated with investment risk, while leverage and firm size show a negative relationship. [8] explored the predictability of financial failure of commercial banks in Pakistan using the Altman Z-score Model. The results of the study show that Altman’s Z-Score model and current ratio appropriately predict the financial failure of commercial banks in Pakistan. [9] also employed the Altman Z-score approach to analyse the financial stability of textile firms using the data for the period 2013–2014–2019–2020. Their results show that textile firms in Pakistan can improve their financial performance, as most of the firms lie in the grey zone according to the Z-score. [10] examined the relationship between investment risk, leverage, and profitability of firms, and concluded that lower investment risk is associated with higher profitability, while higher leverage can negatively impact profitability. [11] identify the financial ratios that are most significant for bankruptcy prediction in the non-financial sector of Pakistan based on a sample of companies that became bankrupt during the time period 1996–2006. Studies in Pakistan have also investigated the relationship between working capital management and profitability [[12] and [13]], as well as the predictive power of market-based metrics in detecting investment at risk [14].

The studies on Pakistan, though, provide valuable insights into investment risk prediction. These studies, however, primarily focus on traditional measures of profitability and firm-specific characteristics. Limited consideration has been given in the literature to external factors such as macroeconomic and political conditions.

Furthermore, the literature lacks comprehensive analytical frameworks that can integrate both internal (firm-level characteristics) and external factors (macroeconomic, financial, and political conditions) to predict investment risk. Literature also ignored the endogeneity issue. Investment risk prediction often involves dynamic relationships in which the current values are influenced by their own past values, as well as by other financial and economic variables, influencing each other, creating an endogeneity issue. The use of robust dynamic panel approaches to address endogeneity in Pakistan’s corporate landscape is also an area that requires further research. Overall, the assessment of investment risk channels in Pakistan remains a relatively under-researched area, signalling the need for broader analyses.

Therefore, this research first aims to assess investment risk using the Altman Z-score, a diagnostic approach. Secondly, it aims to provide robust empirical evidence by applying an integrated investor risk assessment model, an analytical approach that combines firm-specific characteristics, economic conditions, and political stability to assess investment risk. This integrated approach enables a thorough understanding of complex issues, facilitating informed decision-making. The economic model developed is comprehensive, logical, and thus reliable, as it is backed by economic and financial theories and firm-level data (from around 443 firms) for the past 10-year period. The study employs the robust dynamic panel GMM approach to specifically address the endogeneity issue to provide reliable answers to the following questions:

- How does the Altman Z-score, which comprises Leverage, Solvency, Liquidity, and Profitability ratios, instead of employing traditional ratios (such as return on assets, return on equity, and debt to equity ratio), effectively determine investment risk in Pakistan?
- Which among the selected ratios effectively keeps the firms in a risk-free environment or in financial distress?
- Do the firms’ internal factors significantly determine investment risk in Pakistan?
- Which economic and political indicators most significantly impact investment risk in Pakistan?

The first research question is assessed by developing an Investment Risk Index (IR) by employing Altman Z-score, which is a diagnostic tool (as said above) to predict the Investment risk faced by the firms. This will enhance the literature available in the case of Pakistan, assessing the investment risk. The other research questions, focusing on the determinants of the investment risk, are assessed by developing an analytical model based on a thorough review of the literature. The generalized method of moments (GMM) approach has been employed to assess the developed model. Furthermore, to estimate the investment risk model, the data used is extracted from the Financial Statement Analysis of Non-Financial Companies Listed on the Pakistan Stock Exchange (PSX) for the period 2014–2023. The designed analytical model, in conjunction with the micro and macro datasets employed, facilitated the examination of past trends and the investigation of consistent patterns, correlations, and dynamics.

This study advances investment risk assessment literature in Pakistan by extending traditional financial ratio analysis to a Z-score-based Investment Risk Index. Empirical evidence shows the Index averages 5.35, reflecting vulnerabilities in liquidity (0.047), leverage (−5.43), and profitability (−0.005), yet a strong market value of equity suggests lower investor risk. The finding also highlights crucial factors like political stability, GDP growth, and firm-specific characteristics that significantly shape investment risk, offering new insights for risk management in the context of Pakistani firms. These insights can, hence, guide investors and policymakers in mitigating risk and fostering a stable investment environment in Pakistan.

The paper is structured as follows: the next section discusses the analytical approach to identifying investment risk and the factors that can influence it, providing a comprehensive framework for assessing investment risk. Section 2 also provides details of the economic model developed to assess the investment risk and discusses the econometric technique employed. Section 3 presents a descriptive analysis of the data, while Section 4 provides an empirical evaluation of the developed model. The final section concludes and provides policy suggestions.

## 2. IR assessment framework – Analytical approach

**Fig 1:**
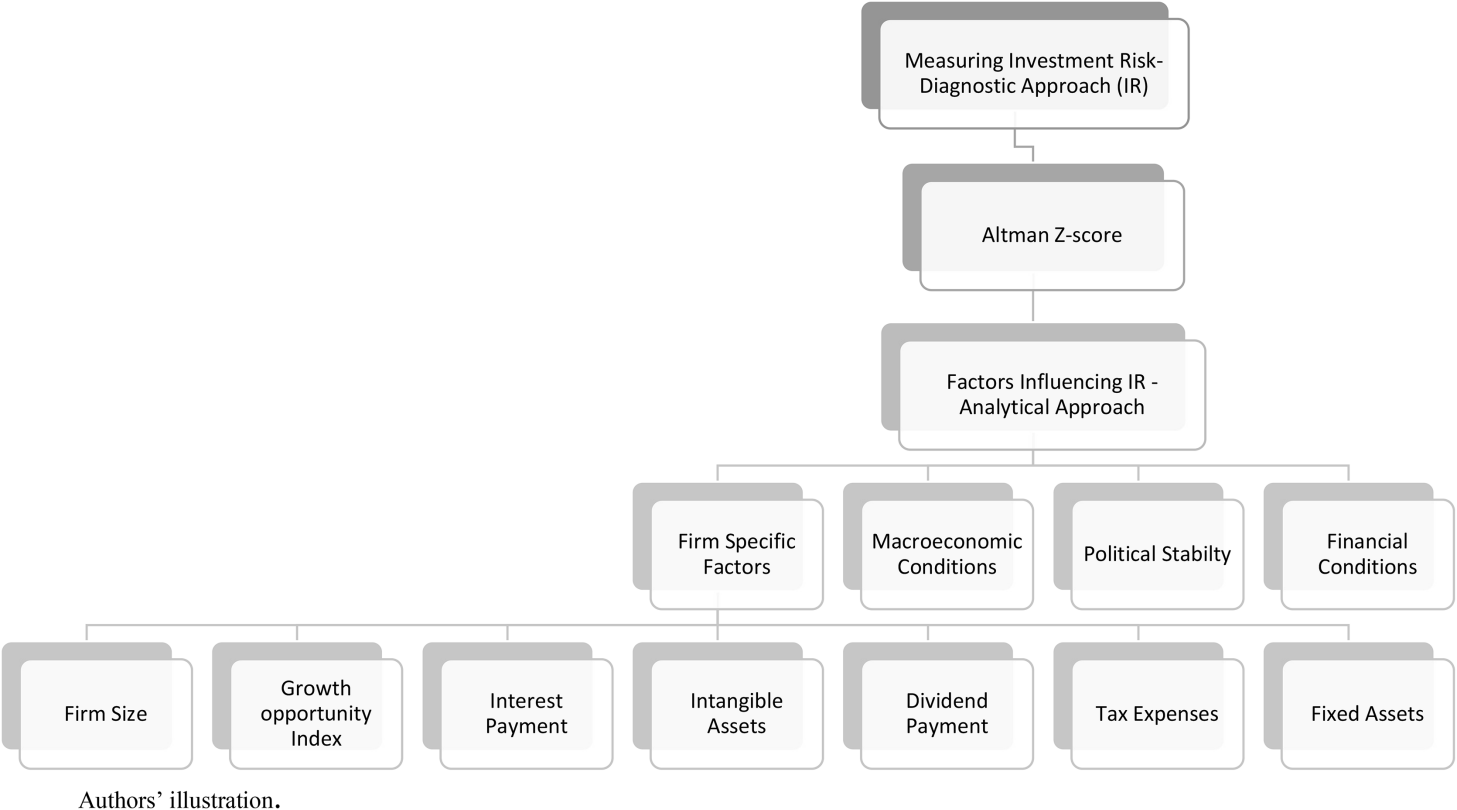
Analytical approach.

The analytical approach to assessing the factors influencing investment risk is presented here using two types of frequently available datasets. The first one focuses on firm-level indicators (available over time) that explain investment risk. In contrast, the second one is based on macro-level data, usually available by time, which is also important for explaining the investment decision. Firm performance is influenced by macroeconomic conditions, and firm productivity and growth are determined by many micro-level factors (firm-specific characteristics). The model is hence developed to explore the contribution of each factor determining investment risk – see Fig 1 for details.

### 2.1 Identification of investment risk

As illustrated above, the assessment is designed based on an extensive literature review. The literature reviewed has provided the knowledge regarding the available instruments for gauging investment risk and the techniques for measuring it. Furthermore, factors influencing investment risk have also been identified through this review. This helped in developing a theoretically strong econometric model to explore the contribution of each identified factor in increasing or decreasing the investment risk.

#### a. Investors’ risk index - IR.

Several variables could explain investors’ risk at the firm level. For example; (i) volatility showing fluctuation in the firm’s stock price or returns, which can indicate uncertainty and risk; (ii) leverage showing the level of debt financing, used by the firm, which can increase the risk of default; (iii) liquidity measuring the ease with which investors can buy or sell shares of the firm’s stock, with lower liquidity increasing risk; (iv) profitability, a strong indicator showing firm’s ability to generate earnings compared to its expenses, with lower profitability indicating higher risk; (v) size, here means market capitalization or asset size of the firm, with smaller firms often considered riskier; (vi) corporate governance measures the quality of the firm’s management, board, and shareholder structure, with weaker governance indicating higher risk. Further, the investment risk score calculates a firm’s likelihood of bankruptcy or financial distress, which is often measured by Altman’s Z-score.

Though a number of indicators in addition to the one explained above are indicated in the literature, after an in-depth review, the study decided to measure the firm’s likelihood of bankruptcy, financial distress or investment risk by calculating Altman’s Z-score, given the findings of [15]. Further, [16] performed a comparison of available prediction models and found that there is no marked difference, in terms of prediction accuracy, between traditional statistical models and more advanced models such as neural networks. This is consistent with the argument given by [17], that there are no unifying agreements between researchers on which model has the highest predictive accuracy. In the case of Pakistan, [15] concluded that the predictive ability of all traditional investment risk prediction models decreased during the period of financial crisis. They concluded that traditional investment risk prediction models, such as the Altman Z-Score Model and the Zmijewski Model, can be relied upon by practitioners in the case of Pakistan.

The Altman Z-score, a variation of the traditional Z-score in statistics, is based on five financial ratios. It uses profitability, leverage, liquidity, solvency, and activity ratios to predict whether a company has a high probability of becoming insolvent, a state of financial distress in which a firm is unable to pay its debts. The original model was based on the firm’s market value and was thus applicable only to publicly traded companies. [18] and [19] advocated a complete re-estimation of the model by excluding the activity ratio to avoid the potential industry effect. Given the broader scope of the revised Z-score, as it is intended for both privately held and publicly listed firms and for both manufacturing and non-manufacturing firms, we based our empirical assessment on the revised Z-score as given in equation 1:


$$\begin{array}{c} \text{Altman Z}-\text{Score}=\text{3}.\text{25}+\text{6}.\text{56}*\text{A}+\text{3}.\text{26}*\text{B}+\text{6}.\text{72}*\text{C}+\text{1}.\text{05}*\text{D} \end{array}$$ (1)

Where:

A = Liquidity = working capital/ total assets (WC/TA)

B = Leverage = retained earnings/ total assets (RE/TA)

C = Profitability = earnings before interest and tax payment/ total assets (EBIT/TA)

D = Solvency = market value of equity/ total liabilities (MVE/TL)

Within the Altman Z-score, solvency ratios indicate a firm’s ability to meet long-term obligations and avoid risk; liquidity ratios, on the other hand, evaluate a firm’s ability to meet short-term obligations and manage working capital. Leverage, though, is more relevant to financial firms, as it relies heavily on debt financing. However, it is worth measuring the investment risk of non-financial firms based on their leverage ratio. According to Altman, a score below 1.1 means the firm is heading for bankruptcy, while firms with scores above 2.6 are enjoying profit, see Table 1 for interpretation of the Z-score values. Investors looking at the average Altman Z-scores of the firms associated with the industry can determine whether they should invest or not in that industry, considering the firms’ underlying financial strength associated with the industry. Recently, the range indicates that a Z-score closer to 0 indicates a firm is in financial trouble. Professor Altman in (2019), in a lecture on “50 Years of the Altman Score,” clarified that 0—not 1.8—is the figure at which investors should worry about a Firm’s financial strength. After identifying the firms’ scores in an industry, the scores of all the firms are averaged to present an industry value.

**Table 1:** Interpretation of the Z-score model.

Z Score	Indicator	Remarks
Z > 2.6	Safe Zone	The firm has good financial stability, and there is minimal chance that it will go bankrupt.
1.10 < Z < 2.6	Gray Zone	The risk of the firm facing financial difficulties in the near future is low
Z < 1.10	Distress Zone	The firm is likely to experience financial challenges in the near future

Note: For details, see [19].

### 2.2 An analytical approach to predict factors influencing investment risk

As stated earlier, the firm’s profitability is influenced by the overall economy’s performance, political stability, and financial position (external factors), and firm-specific characteristics (internal factors). Our economic model evaluates investment risk and, hence, takes into account both internal and external factors (see S4 Table in supporting information for variables description). The sub-sections below discuss each indicator included in the final model in greater detail, highlighting their importance and impact.

#### b. Investor’s risk assessment based on economic, political and financial conditions - External factors.

Various macroeconomic factors, including GDP growth, inflation rates, interest rates, and government fiscal policies, significantly influence a firm’s financial health. Investors carefully assess these factors to evaluate the overall economic environment and associated risks. Literature highlighted that a strong GDP growth rate typically indicates a healthy economy, while high inflation or interest rates may signal economic instability, impacting investment returns. Furthermore, the integration of the financial system with international markets adds an additional layer of risk assessment, as global economic conditions can also impact local investments.

Given the argument that investors must stay informed about macroeconomic indicators to effectively manage risks, this study identifies a list of variables showcasing macroeconomic performance, political stability, and the country’s financial condition as potential determinants of Investment Risk (IR). These variables include GDP per capita, GDP growth rate, annual inflation rate, broad money, and current account balance.

In this study, these variables are used in alternative models to identify the most significant determinant of IR. The final model uses the GDP growth to evaluate the impact of a country’s economic performance on IR. A high GDP growth rate is expected to have a positive effect on IR, as it implies high economic activity, essential for lowering default risks associated with investment. Inflation is also crucial, as high inflation can lead to inflationary risk. Understanding the inflation dynamics helps investors gauge potential returns against the erosion of purchasing power and adjust their strategies accordingly.

Political stability is also essential for investment decisions. A stable political environment fosters robust economic growth, increasing investor confidence and boosting investment decisions. The model includes an index of political stability, and expects that the political stability will reduce IR.

In addition to macroeconomic performance and political stability, the assessment also includes an indicator of monetary policy. Broad money growth is used to evaluate the country’s financial condition. A higher percentage may indicate more liquidity, stimulating economic activity but also raising concerns about inflation and financial stability.

Finally, the impact of the current account balance is also included in the model, as it impacts the investment climate by influencing factors such as trade balance, foreign exchange stability, debt obligations, and overall economic health. Hence, it may reduce IR. Pakistan’s persistent current account deficit erodes investors’ confidence as investors perceive the country’s economy as weak or unstable.

#### c. Investor risk assessment based on firm-specific characteristics – Internal factors.

Investment decisions are influenced by various firm-level characteristics, as well, which also affect firm performance. Firm-specific characteristics are expected to interact with each other and with macroeconomic factors to influence investors’ risk perception. In addition to external factors, these indicators are also explored here to investigate their effect on investment risk.

Among the financial ratios used to compute the investment risk index (measured by employing the Altman Z-score approach), liquidity, leverage, and productivity positively affect firm performance. At the same time, solvency and asset turnover are positive and statistically significant only in the case of return on equity [20]. Further, firms with a low leverage ratio – the proportion of retained earnings to total assets- mainly finance their capital expenditure through borrowings. Several factors can influence retained earnings, with profitability and dividend policy being two key drivers. Moreover, for a firm to be profitable, it must have a higher asset turnover ratio and hold liquidity if it faces financial friction.

Further, Solvency refers to a firm’s ability to meet its long-term obligations, including interest and principal debt. A high solvency ratio indicates a firm is at a low risk of bankruptcy, while a low solvency ratio means a firm is at a higher risk of bankruptcy. A firm can improve its Solvency Ratio by reducing its debt or increasing its equity. The low debt-to-equity ratio, a commonly used ratio that measures a firm’s leverage, indicates that a firm has lower debt than equity and will definitely improve the solvency ratio. This objective can be achieved by paying off debt, issuing new equity, or retaining earnings.

In addition, a firm uses its fixed, long-term assets to produce goods or services. A higher proportion of fixed assets held by the firms indicates higher productivity, ensuring a higher asset turnover ratio. Fixed assets are costly and require maintenance and depreciation; hence, operating fixed assets after deducting accumulated depreciation is preferred in the model. A firm should ensure that its fixed assets are utilised efficiently and effectively to have a higher asset turnover ratio. Further, liquidity, profitability and solvency ratios are all assumed to affect the asset turnover ratio significantly.

Based on the intensive review and the discussion above, factors that could best determine the investors’ risk are discussed in Table 2 below:

**Table 2:** Variables relationship with investment risk.

Variables	Relationship with Investment Risk [Note: a higher value of the Investment Risk (calculated using Altman Z-Score Approach) indicates financial stability (reduces risk)]	Expected Sign
GDP Growth	A higher GDP growth rate indicates a healthy economy, impacting investment returns positively; hence, investment risk declines with the increase in GDP growth.	-ve
Political Stability	A stable political environment fosters robust economic growth, increasing investor confidence and boosting investment decisions, thus political stability also reduces investment risk.	-ve
Inflation Rate	High inflation signals economic instability, impacting investment returns negatively. Conversely, a positive relationship between inflation and investment risk is also evident from the literature, which indicates that firms may enjoy the inflationary environment by increasing prices, potentially leading to higher revenues.	-ve/ + ve
Current Account Balance	The current account balance impacts the investment climate by influencing factors such as trade balance, foreign exchange stability, debt obligations, and overall economic health. Any improvement in the current account balance means export expansion and thus increases investment opportunities and revenues. Therefore, investment risk usually declines with the improvement in the current account balance.	-ve
Broad Money	Investor risk perception is shaped by the stability of the financial system and the effectiveness of monetary policy in managing liquidity. As money becomes cheaper and more accessible, firms may take on more debt to finance operations and growth. While this can initially boost returns, it increases risk in the long run.	-ve/ + ve
Dividend Payment	Dividend payments are a cash outflow. This reduces a firm’s retained earnings and, therefore, impacts its financial position. Paying out a significant portion of earnings as dividends results in less money available to reinvest in the business, potentially reducing firms’ growth prospects. Firms with high dividend payouts are more vulnerable to financial distress and face high investment risk. Contrary to this, the impact could be positive because when firms distribute a high portion of their profits to shareholders in the form of dividends, it sends a strong signal about the company’s financial health, stability, and commitment to rewarding investors.	-ve/ + ve
Interest Payment	A high Interest payment means a firm has a high level of debt, hence a low level of profitability. Firms with high interest payments and limited cash flow are at risk of defaulting on their debt obligations, which can lead to significant financial losses for investors.	+ve
Tax expenses	Tax expenses are again a cash outflow, reducing a firm’s retained earnings and potentially impacting its financial flexibility. A high tax expense can reduce a firm’s profitability, making it more challenging for investors to generate returns. Conversely, higher tax payments by a firm mean substantial profitability enjoyed by the firm, boosting investors’ confidence as tax liabilities are a function of earnings.	-ve/ + ve
Operating Fixed Assets	Firms operating fixed assets can serve as tangible collateral, reducing the risk of lending to the firm and potentially decreasing investment risk. Firms with large fixed assets can benefit from economies of scale, increasing efficiency and reducing costs, which can decrease investment risk. Conversely, higher asset tangibility though can raise collateral value but it also increase burden on firms. The burden of fixed costs and depreciation, potentially increases the default risk.	-ve/ + ve
Growth opportunities [given by; Book value per share (market-to-book ratio)]	Growth opportunities measured through book value per share can have both positive and negative effects on investment risk. While a high book value per share can indicate financial flexibility and reduced financial leverage, it may also signal overvaluation, asset intensity, or unrealistic growth expectations, increasing investors’ risk	-ve/ + ve
Firm Size [Measured through the natural logarithm of the mean of the total assets, i.e., FS = log (total assets)]	Impact can be both positive and negative. Research suggests that there is a negative relationship between firm size and investment risk. Larger firms tend to have (i) more diversified revenue streams, reducing their exposure to market volatility and (ii) more financial resources, enabling them to better absorb shocks and respond to changing market conditions. Contrary to the above, large firms also require significant investments in infrastructure and tie up substantial capital, making them vulnerable to economic shocks. Increased regulations also elevate the investment risks associated with large-scale investments.	-ve/ + ve

### 2.3 Presence of endogeneity

Economic theory strongly indicates that the lagged value of the dependent variable is predetermined in the model, and the control variables are mainly endogenous. Hence, a dynamic panel data model using the Generalized Method of Moments (GMM) is employed here (see section 2.4 for details). GMM account for the potential lagged effects of Investment risk. The lagged dependent variable, hence, is used as an explanatory variable to evaluate the effect of its past values on determining current investment risk.

Further, in a panel data model assessing investment risk, using the log of total assets as a proxy for firm size can be problematic due to potential endogeneity. Endogeneity may arise as unobserved factors simultaneously affect firm size and the measured investment risk. This could lead to biased estimates and incorrect inferences. Therefore, it is essential to consider the possibility of endogeneity and apply appropriate econometric techniques to address this issue.

The same is true for growth opportunities measured through book value per share. A firm’s book value per share is endogenous because various firm-specific factors, such as size, risk level, and management decisions, influence its investment policies and book value per share. Additionally, the firm’s strategies and external market conditions could dynamically affect its perceived value, creating endogeneity in the relationship being studied.

In addition, investment risk is interdependent on dividends, interest, and tax payments. These payments imply that not only is investment affected by them, but the decision to pay these expenses reflects the firm’s financial condition, making these factors endogenous in the model as well.

Firms’ holdings of fixed operating assets can also be endogenous. Endogeneity arises because fixed operating assets influence productivity and overall firm financial performance, which may, in turn, affect investment decisions and, hence, risk assessments.

Overall, it can be concluded that investment risk modelling involves dynamic relationships where its current values are influenced by its own past values, as well as by other financial and economic variables, influencing each other, creating an endogeneity issue. This means that the explanatory variables in the regression model will be correlated with the error term, leading to biased and inconsistent estimates if not handled properly. GMM, specifically system GMM, is preferred here to handle the issue. System GMM is preferred over Difference GMM as it estimates the model simultaneously in both differences and levels, providing more robust results, especially for a dynamic panel data model with persistent explanatory variables, as in this case.

### 2.4 Econometric estimation

This study employs a dynamic panel data model to investigate the relationship between variables. Traditional estimation methods, such as OLS and random effects, yield biased results due to the presence of lagged dependent variables. The lagged dependent variable needs to be treated as a regressor on the right-hand side, as it shows some degree of persistence in the level of Investment Risk (IR). Past studies, therefore, encourage the design of a dynamic panel model for the consistent estimation of parameters, indicated in equation 2.


$$\begin{array}{c} {IR}_{it}=\alpha {IR}_{it-\text{1}}+\beta X_{it}+\eta_{i}+\varepsilon_{it}\;|\alpha |<\text{1},\;i=\text{1}\ldots \ldots .N\;\&\;t=\text{1}\ldots .\;T \end{array}$$ (2)

Where subscripts i and t denote the cross-sectional and time dimension of the panel, respectively. $X_{it}$ is the vector of independent variables concerning macroeconomic, political, financial, and firm-specific factors assumed to be affecting IR other than the lagged IR. α and β are the vectors of coefficients to be estimated. $\eta_{i}\;$ is the unobserved firm-specific effect, and $\varepsilon_{it}$ is the error term. Equation (2) assumes that the error term $\varepsilon_{it}$ satisfies the orthogonality conditions. In Equation (2), ${IR}_{it-\text{1}}$ is correlated with the fixed effects, which is called the dynamic panel bias that cannot be solved by the static panel data models.

Specifically, in the presence of a lagged dependent variable, ordinary least square estimation gives upward-biased results. This research utilises the Generalized Method of Moments (GMM) framework to address the issue. This method effectively mitigates dynamic panel bias and endogeneity concerns. The generalized method of moments (GMM) proposed by [21] and generalized by [22] is more efficient in solving the dynamic panel bias. The estimators address such problems by first differencing Equation (2) as follows:


$$\begin{array}{c} {\Delta IR}_{it}=\alpha {\Delta IR}_{it-\text{1}}+\beta \Delta X_{it}+{\Delta \varepsilon }_{it} \end{array}$$ (3)

Equation (3) removes the fixed effect, but the lagged dependent variable is still correlated with the new error term. GMM estimations also solve such endogeneity problems. Both difference GMM and system GMM are designed to remove the dynamic panel bias [21] & [23] through instrumental variables.

The System GMM estimator employs instrumental variables to address endogeneity, ensuring the validity and exogeneity of the instruments. To verify the appropriateness of the GMM specification, this study conducts two diagnostic tests:

1. The Sargan test evaluates the validity of the instruments.
2. The serial correlation test assesses the assumption of serially uncorrelated errors.

Additionally, GMM is specifically designed for a short time dimension with a large number of cross-sectional panels, and where all the independent variables are not strictly exogenous. It is precisely the case in our sample where T = 10 and N = 443. For the reliable estimation results, we ensure that the number of instruments does not exceed the number of cross-sections (firms) over the study period.

### 2.5 Identification of datasets

The firm-level dataset is extracted from audited “Financial statements of non-financial companies listed at Pakistan Stock Exchange” published by the State Bank of Pakistan annually [24]. For the assessment purpose, the study extracted data for around 443 firms for the period 2014–2023. While the Macro-level variables are obtained from the World Development Indicators published by the World Bank. The observed variables for computing political risk components are extracted from the Freedom in the World (FIW) dataset. Economic risk and financial risk indicators are available in the State Bank of Pakistan’s (SBP) annual reports (downloadable from: https://www.sbp.org.pk/reports/annual/FSANFC/Years.htm). All the statistics are extracted annually from 2014 to 2023 to maintain the coherence between firm-level and macro-level assessments. The firm-level data and macro-level data are consolidated using time as a connecting variable. The data used in the study are secondary and are publicly available.

## 3. Assessment of firm-level data

The highly balanced panel of around 443 firms is retrieved from the databank of the State Bank of Pakistan published in 2023 [24]. Table 3 shows that the textile sector’s share is highest in the data, around 35%, followed by the Chemicals, Chemical Products, and Pharmaceutical Sector’s share, around 11.5%. The manufacturing sector accounts for a 9.5% share of the total sample. The other sector having more than 5% share in the sample includes: Sugar Sector (7%), Fuel and Energy Sector (5.2%), Food Sector (5.2%) and Motor Vehicles, Trailers & Auto Parts Sector (5.2%). The overall sample size is 4430 (consisting of around 443 firms for a 10-year period).

**Table 3:** Share of firms in the total sample.

Sector	2014	2015	2016	2017	2018	2019	2020	2021	2022	2023	Share in Total
Cement Sector	20	20	20	20	20	20	20	20	20	20	4.5
Chemicals, Chemical Products And Pharmaceutical Sector	51	51	51	51	51	51	51	51	51	51	11.5
Coke And Refined Petroleum Products	11	11	11	11	11	11	11	11	11	11	2.5
Electrical Machinery And Apparatus	9	9	9	9	9	9	9	9	9	9	2.0
Food Sector	23	23	23	23	23	23	23	23	23	23	5.2
Fuel And Energy Sector	23	23	23	23	23	23	23	23	23	23	5.2
Information And Communication Services	22	22	22	22	22	22	22	22	22	22	5.0
Manufacturing Sector	42	42	42	42	42	42	42	42	42	42	9.5
Mineral Products	9	9	9	9	9	9	9	9	9	9	2.0
Motor Vehicles, Trailers & Auto Parts Sector	23	23	23	23	23	23	23	23	23	23	5.2
Other Services Activities	12	12	12	12	12	12	12	12	12	12	2.7
Paper, Paperboard And Products	10	10	10	10	10	10	10	10	10	10	2.3
Sugar Sector	31	31	31	31	31	31	31	31	31	31	7.0
Textile Sector	157	157	157	157	157	157	157	157	157	157	35.4
Total	443	443	443	443	443	443	443	443	443	443	4430

Source: Authors’ Illustration based on the Data Extracted from “Financial statements of non-financial companies listed at Pakistan Stock Exchange”.

Note: The State Bank of Pakistan’s financial statements analysis for non-financial firms is heavily reliant on data from firms listed on the Pakistan Stock Exchange (PSX), where textile firms have a significant presence. Consequently, the analysis has a higher representation of textile firms, and a substantial proportion of the sample consists of companies from this sector.

### 3.1 Assessment of Altman Z-score – Investment risk index

Table 4 below provides the descriptive statistics of the ratios used in constructing the Investment Risk Index. Overall, the Index’s average value is 5.35, while the average liquidity ratio value is 0.047, leverage is −5.43, profitability is −0.005, and solvency is 1.692. A low value of the liquidity ratio indicates that the sample firms, on average, might be struggling to meet their short-term debts and hence may be subject to bankruptcy. A negative value of the leverage ratio further signifies that, on average, sample firms’ debt obligations outweigh their ability to generate returns, potentially leading the investment to risk. Further, an average value less than 1 for the earnings before tax (EBT) over total assets ratio (profitability ratio) indicates that the sample firms’ earnings before tax are lower than their total assets. This suggests potentially lower profitability relative to their asset bases. Contrary to all other indicators, the average market value of equity is significantly higher than its total liabilities. This suggests a strong financial position of the sample firms and potentially lower risk for investors.

**Table 4:** Descriptive statistics of the ratios used for constructing Altman Z-scores.

Variable		Mean	Min.	Max.	Std. Dev.
Working Capital Over Total Assets	Overall	0.047	0.000	0.921	0.091
	Between		0.000	0.656	0.074
	Within		−0.608	0.654	0.053
Retained Earnings Over Total Assets	overall	−5.433	−1699.827	0.834	66.731
	between		−956.400	0.723	58.721
	within		−1385.219	311.773	31.816
Earnings Before Investment And Taxes Over Total Assets	overall	−0.005	−76.214	366.204	7.190
	between		−72.698	35.709	3.890
	within		−79.898	330.491	6.050
Market Value Of Equity Over Total Liabilities	overall	1.692	−1.195	72.759	5.011
	between		−1.010	71.551	4.773
	within		−30.304	33.904	1.542
Altman Z-Score	overall	5.350	−202.493	47.021	5.522
	between		−23.624	35.220	4.050
	within		−173.520	42.530	3.760

Source: Authors’ Illustration based on the Data Extracted from “Financial statements of non-financial companies listed at Pakistan Stock Exchange”.

The minimum values in thousands for Retained Earnings and EBIT over total assets could be considered as outliers, but the exclusion of such values might lead to the exclusion of firms struggling to survive. This creates an overly optimistic view of market performance and may lead to inaccurate conclusions. Considering that the research specifically investigates investment at risk, or bankruptcy, it requires the inclusion of firms struggling to survive as well. They are central to understanding the causes and consequences of poor financial health.

### 3.2 Investment risk and indicators contributing to investment risk

Table 5 assesses the proportion of firms in various risk zones. The division is based on the [19] benchmarks, as indicated in Table 1. The assessment shows that the proportion of firms in the distress zone increased from 4.8% to 8.5% (almost doubled) while the proportion of firms in the grey zone first increases from 3.3% to 6.6%, but then it again declines to 3.3%. The proportion of firms in the safe zone declined by 2% during the period 2014–2023. This shows a mixed picture of the financial stability of the sampled firms during the period.

**Table 5:** Distribution of sample firms by distress Zone.

	Distress Zone		Grey Zone		Safe Zone	
2014	16	4.8%	11	3.3%	304	91.8%
2015	21	6.3%	13	3.9%	297	89.7%
2016	23	6.9%	17	5.1%	291	87.9%
2017	23	6.9%	17	5.1%	291	87.9%
2018	28	8.5%	16	4.8%	287	86.7%
2019	30	9.1%	13	3.9%	288	87.0%
2020	30	9.1%	22	6.6%	279	84.3%
2021	30	9.1%	16	4.8%	285	86.1%
2022	28	8.5%	11	3.3%	292	88.2%
2023	28	8.5%	11	3.3%	292	88.2%
Total	257	7.8%	147	4.4%	2906	87.8%

Source: Authors’ Illustration based on the Data Extracted from “Financial statements of non-financial companies listed at Pakistan Stock Exchange”.

Table 6 assesses the ratios that mainly contribute to the sampled firms’ financial performance. In Table 6, retained earnings over total assets remained strongly related to the investment risk throughout the study period. In other words, the poor performance of this indicator has kept the firms in the distress zone. For the firms in the grey zone, almost all the financial ratios show low correlation; hence, for the firms in this zone, we cannot point out any one indicator as a reason for the poor performance, as all are equally contributing. Table 6 also points towards the decisive role of the market value of equity over total liability on firms’ financial performance. This ratio is highly correlated with the investment risk in almost all years (see also supporting information S1–S3 Tables).

**Table 6:** Correlation between the financial ratios and the Z-score.

	Distress Zone				Grey Zone				Safe Zone			
Years	WC/TA	RE/TA	EBIT/TA	MVE/TL	WC/TA	RE/TA	EBIT/TA	MVE/TL	WC/TA	RE/TA	EBIT/TA	MVE/TL
**2014**	−0.07	0.99	−0.19	0.76	0.35	0.45	0.14	0.41	0.14	0.44	0.31	0.93
**2015**	−0.13	0.89	−0.01	0.66	−0.06	0.44	0.27	0.17	0.34	0.33	0.21	0.95
**2016**	0.17	0.81	0.07	0.23	−0.35	0.61	−0.21	0.31	0.15	0.46	0.35	0.9
**2017**	−0.16	0.97	−0.11	0.66	0.11	0.13	0.17	0.32	0.15	0.45	0.33	0.89
**2018**	0.04	0.94	0.23	0.7	0.04	0.55	−0.53	−0.17	0.1	0.48	0.29	0.9
**2019**	−0.16	0.9	0.26	0.69	−0.47	0.45	0.42	−0.01	0.06	0.41	0.21	0.92
**2020**	−0.04	0.98	0.27	0.54	−0.01	0.42	−0.17	0.38	0.06	0.32	0.18	0.92
**2021**	−0.06	0.99	−0.9	0.06	−0.31	0.4	0.08	0.41	0	0.4	0.18	0.93
**2022**	−0.07	0.99	−0.14	0.5	−0.46	0.67	0.38	−0.27	0.03	0.41	0.15	0.92
**2023**	−0.03	0.99	0.02	0.55	−0.07	0.43	−0.33	0.53	0.02	0.37	0.25	0.89
Note:	**Strong**	**Moderate**	**Weak**	**Poor**								

Source: Authors’ Illustration based on the Data Extracted from “Financial statements of non-financial companies listed at Pakistan Stock Exchange”.

Overall, the firms associated with the textile products not classified specifically (other textile n.e.s) have shown poor financial performance during the period, based on the Altman Z-score (with a Z-score value 2.4), which suggests that investors should make cautious decisions about investing in this sector. Table 7 provides the breakdown of the investment risk scores by sector. Table 8 further shows that around 8% of the sampled firms were in the distress zone, 4% in the grey zone, and 88% in the safe zone during the period.

**Table 7:** Investment risk by sector.

Sub Sector	IR – Average Score
Cement	6.4
Chemicals, Chemical Products and Pharmaceuticals	5.5
Coke and Refined Petroleum Products	5.2
Electrical Machinery	4.6
Food	6.7
Fuel and Energy Sector	6.4
Information and Communication	7.6
Made-up textile articles	4.8
Manufacturing	6.0
Mineral products	4.7
Motor Vehicles, Trailers and Auto parts	5.5
Other Services Activities	7.6
Other textiles n.e.s.	2.4
Paper, Paperboard and Products	6.4
Spinning, Weaving, Finishing of Textile	4.2
Sugar	5.0

Source: Authors’ Illustration based on the Data Extracted from “Financial statements of non-financial companies listed at Pakistan Stock Exchange”.

**Table 8:** Percentage distribution of sampled firms by distress zones.

Zones	Firm Proportion
Distress Zone	8%
Grey Zone	4%
Safe Zone	88%

Source: Authors’ Illustration based on the Data Extracted from “Financial statements of non-financial companies listed at Pakistan Stock Exchange”.

Table 9 shows the percentage distribution of firms by sectors and zones. It is evident from the table that the firms associated with the other textile sector n.e.s have faced more risk during the study period.

**Table 9:** Percentage distribution of sampled firms by sector and distress zones.

	Distress Zone	Grey Zone	Safe Zone
Cement	5.56	1.11	93.33
Chemicals, Chemical Products and Pharmaceuticals	5.41	1.62	92.97
Coke and Refined Petroleum Products	5.45	5.45	89.09
Electrical Machinery	7.5	5	87.5
Food	3.16	2.11	94.74
Fuel and Energy Sector	5.56	0	94.44
Information and Communication	20	2.86	77.14
Made-up textile articles	0	0	100
Manufacturing	0	1.82	98.18
Mineral products	12.5	1.25	86.25
Motor Vehicles, Trailers and Auto parts	6	5	89
Other Services Activities	1.11	4.44	94.44
Other textiles n.e.s.	35	2	63
Paper, Paperboard and Products	0	2.86	97.14
Spinning, Weaving, Finishing of Textile	11.26	8.62	80.11
Sugar	5.17	7.24	87.59

Source: Authors’ Illustration based on the Data Extracted from “Financial statements of non-financial companies listed at Pakistan Stock Exchange”.

## 4. Empirical results based on GMM

Table 10 presents the results of panel regression using the GMM specification. The first and second columns show the model analysing the impact of economic, political and financial indicators of the economy on deriving the investment risk, the external factors affecting the firms’ profitability. Exclusively, Column 1 incorporates the GDP growth, while GDP per capita replaces the GDP growth in Column 2. Column 3 shows the impact of a firm’s internal factors on the investment risk index. While Column 4 shows the results of both external and internal factors on the investment risk.

**Table 10:** Factors contributing to IR.

	Column 1		Column 2		Column 3		Column 4	
	Coef.	P > |z|	Coef.	P > |z|	Coef.	P > |z|	Coef.	P > |z|
Log of Lag 1 of IR	0.734	0.00*	0.762	0.00*	0.412	0.00*	0.395	0.00*
Political Stability Index	0.022	0.18	0.102	0.00*			0.032	0.00*
GDP Growth	0.014	0.00*					0.008	0.00*
Log of GDP Per Capita				−0.008	0.00*			
Log of GDP Per Capita Square			5.75	0.00*				
Inflation, consumer prices (annual %)	0.001	0.34	−0.004	0.00*			0.003	0.00*
Current account balance (% of GDP)	0.009	0.00*	−0.011	0.12			0.010	0.00*
Broad money growth (Annual %)	0.000	0.90	−0.009	0.00*			−0.003	0.01*
Log Dividend Paid					0.027	0.00*	0.031	0.00*
Log Operating Fixed Assets					−0.024	0.00*	−0.015	0.00*
Log Interest Payment					−0.026	0.00*	−0.033	0.00*
Log Tax Expenses						0.012	0.00*	−0.001
Firm Size					−0.011	0.10**	−0.032	0.00*
Log of Growth Opportunities					0.049	0.00*	0.040	0.00*
Constant	0.48	0.00*	−71.05	0.00*	1.196	0.00*	1.852	0.00*
Number of obs	2767		2767		992		992	
Number of groups	320		320		215		215	
Number of instruments	61		61		141		157	
Wald chi2 Test	2216.78	0.00*	2332.23	0.00*	2618.57	0.00*	3694.65	0.00
Sargan Test	93.86	0.00*	80.46	0.01*	144.01	0.24	149.32	0.36
AR (1)	−5.67	0.00*	−5.81	0.00*	−4.22	0.00*	−4.48	0.00
AR (2)	0.40	0.69	0.087	0.93	−0.46	0.65	−0.57	0.57

Source: Author’s Illustration.

Before going into the discussion, the study confirms the econometric specification’s validity. For this purpose, the study has employed the Arellano-Bond test to check that error terms are not serially correlated. The p-value confirmed that there is no second-order serial correlation in the error terms, i.e., all the models passed the AR (2) tests. Further, the number of instrumental variables in all the specifications is less than the number of firms and the validity of the instruments is confirmed by performing the Sargan test. Overall, the p-values of the Sargan test validate that the instruments employed for all the models follow over-identifying restriction assumptions, except the one having only external factors – Macro-economic conditions, political and financial conditions of the economy. This might be because of the restricted valid instruments in the model. Therefore, the study confirmed that the estimated results are adequately specified by considering all the test statistics. Hence, the rest of the section discusses the results with the affirmation that it follows all the assumptions. Among the many specifications of the model presented in Table 10, we have chosen column 4 for the interpretation of the results, as it shows more predictive power of the chosen variables.


$$\begin{array}{cc} {IR}_{it}=\; & \text{1}.\text{8}\text{5}\text{2}+\text{0}.\text{3}\text{9}\text{5}{\left(Lag\;of\;log\;of\;IR\right)}_{it}+\text{0}.\text{0}\text{3}\text{2}{\left(Political\;Stability\;Index\right)}_{it}+\text{0}.\text{0}\text{0}\text{8}(GDP\;Growth{)}_{it}\\ & \;+\text{0}.\text{0}\text{1}(Current\;account\;balance\;\%\;GDP{)}_{it}+{\text{0}.\text{0}\text{0}\text{3}(Inflation\;Annual\;\%)}_{it}-\text{0}.\text{0}\text{0}\text{3}(Broad\;Money{)}_{it}\\ & \;+\text{0}.\text{0}\text{3}\text{1}(Log\;of\;Dividend\;Payable{)}_{it}-\text{0}.\text{0}\text{1}\text{5}(Log\;of\;Operating\;Fixed\;Asset{)}_{it}-\text{0}.\text{0}\text{3}\text{3}(Log\;of\;Interest\;Payment{)}_{it}\\ & \;-\text{0}.\text{0}\text{0}\text{1}{\left(Log\;of\;Tax\;Payment\right)}_{it}-\text{0}.\text{0}\text{3}\text{2}{\left(Firm\;size\right)}_{it}+\text{0}.\text{0}\text{4}{\left(Growth\;Opportunity\right)}_{it} \end{array}$$

As mentioned above, the Altman Z-Score is used here to indicate investment risk. It is worth noting that an increase in the indicator means that the investment risk faced by the firms decreases, and a decrease in the indicator value shows that the investment risk faced by the firms increases, i.e., higher Z-scores imply lower risk. Therefore, the results obtained from the models evaluate whether any independent variable positively affects the Z score by decreasing the investment risk or negatively affects the Z score by increasing the investment risk.

The estimation results confirm that both internal factors (firm-specific characteristics) and external factors (such as economic, political, and financial stability indicators) significantly impact investment risk, either explored separately as in columns 1, 2 and 3 or jointly in columns 4.

Starting with the lagged dependent variable, a highly significant and positive coefficient confirms that investment risk is derived from its past value. The higher the firms’ exposure to investment risk in the past, the higher the probability that they will continue to face the financial risk in the future as well. Specifically, a 1 percent increase in the risk value is associated with a 0.395 percent increase in the future risk value.

Among the explanatory variables, a significant positive coefficient of the political stability index shows that a 1 percent increase in the political stability index increases the Altman Z-scores by 0.032 percent, which means the investment risk decreases by 0.032 percent. Political stability fosters a more predictable and secure business environment, encouraging investment, reducing uncertainty, and promoting economic growth. These factors contribute to improved financial health, as reflected by the positive coefficient of the Political Stability Index. Under stable political conditions, firms can plan for the future with more confidence, access funding more efficiently, and experience less disruption in their operations, all of which affect their financial performance.

A significant positive association between GDP growth and investment risk index confirms that a higher GDP growth rate indicates a healthy and expanding economy, fostering production and thus reducing investment risk. This may foster investor confidence to invest in the economy as well. Specifically, a 1 percent GDP growth increases the index by 0.008 percent, which indicates that investment risk will decline by 0.008 percent if the GDP growth increases by 1 percent.

Though a significant positive impact of GDP growth confirms the hypothesis that in a growing economy, investment risk is lower. However, column 2 indicates a negative but non-linear relationship between GDP per capita and the index developed. The higher GDP per capita first increases the investment risk, but after a certain point/threshold, a further increase will decrease the investment risk.

Further, a positive relationship between inflation and the investment risk index here indicates that sampled firms enjoy the inflationary environment by increasing prices, potentially leading to higher revenues. Also, as the investment risk index is based on leverage, solvency and profitability indicators, a positive impact may also mean a high inflationary period is leading to a reduction in the real value of existing debt and an improvement in the value of the firms’ physical assets. All these enhance firms’ balance sheet and positively influence the index – Altman Z-score in this case. Precisely, a 1 percent increase in the inflation rate increases the Z-score slightly by 0.003 percent thereby reducing the investment risk by the same percent.

The current account balance is found to have a significant positive impact on the Altman Z-score. The current account balance impacts the investment climate by influencing factors such as trade balance, foreign exchange stability, debt obligations, and overall economic health. Pakistan’s persistent current account deficit since its inception has led investors to perceive the country’s economy as weak or unstable, eroding investor confidence. This diminished confidence often affected the valuation indicators used in the calculation of the Altman Z-score. Firms operating in economies with chronic current account deficits, like Pakistan, face long-term financial instability, as these deficits often indicate underlying structural weaknesses. Definitely, any improvement in the current account balance will decrease the risk of bankruptcy and lead to an improvement in the Z-scores. A 1 percent increase in the current account balance is found to improve the Z-score by 0.01 percent, indicating a decline in the investment risk.

In addition to the above explanatory variables, another variable included in the analysis related to monetary policy is broad money as a percentage of GDP. Investor risk perception is shaped by the stability of the financial system and the effectiveness of monetary policy in managing liquidity. The significant negative relationship indicates that the investment risk index decreases by 0.003 percent as the ratio increases by 1 percent, which means firms will face financial instability (investment risk will increase by 0.003 percent when the broad money increases by 1 percent). As money becomes cheaper and more accessible, firms take on more debt to finance operations and growth. While this can initially boost returns but it increases financial risk in the long run. A higher broad money ratio also reflects risky lending practices. Increased broad money could also reflect lending to less productive sectors, decreasing efficiency, and profitability. All this can lead to financial instability for firms.

Among the firm-specific characteristics, the positive coefficient of dividend premium shows that dividend payments have a profoundly positive impact on investor confidence instead of a negative impact. This is possible because when firms distribute a portion of their profits to shareholders in the form of dividends, it sends a strong signal about the company’s financial health, stability, and commitment to rewarding investors. This boosts investors’ confidence and reduces risk. A 1 percent increase in the dividend paid decreases the investment risk by 0.031 percent.

Further, a 1% increase in a firm’s operating fixed assets is found to be associated with a 0.015% decline in the z-score, implying a corresponding 0.015% escalation in investment risk. This finding suggests that investments in fixed assets have a detrimental impact on cash flow, potentially straining a firm’s financial stability and increasing its vulnerability to distress. Specifically, firms with higher fixed assets face higher maintenance, capital expenditure, and regulatory or compliance costs, which can affect liquidity and increase investment risk.

For tax expenses, the results show a negative but insignificant effect of the tax expense; hence, nothing can be firmly concluded.

The negative and significant effect of the Firm Size confirms the belief that larger firms require significant investments in infrastructure, which usually tie up substantial capital, making them vulnerable to economic shocks. Further, increased regulations may also have elevated the investment risks associated with large-scale investments. Precisely, a 1 percent increase in the firm size is found to decrease the Z-score by 0.032 percent, which means it increases the investment risk by 0.032 percent.

Growth opportunities here, measured through book value per share, positively affect investment risk. This indicates that financial flexibility and reduced financial leverage are decreasing investors’ risk by 0.04 percent.

## 5. Conclusion

The study employs the Altman Z-score technique, a diagnostic approach to measure the investment risk index for the firms operating in Pakistan. The study also develops an analytical framework to assess factors influencing Investor Risk in Pakistan by integrating firm-specific characteristics, macroeconomic conditions, and political stability. Further, using a dynamic panel data approach through the Generalized Method of Moments (GMM), the research highlights the significance of both internal (firm-specific) and external (macroeconomic and political) factors in shaping investment decisions.

The results show that the proportion of Firms in the distressed zone increased while the proportion of firms in the grey zone first increases, then declines, during the period of study. The proportion of firms in the safe zone, on the other hand declines during the period 2014–2023. Retained earnings over total assets are found to be strongly related to the investment risk index; poor performance of this indicator leads firms into the distress zone. To keep firms in a safe zone, the Market value of equity over total liabilities remained an important factor, suggesting that higher equity relative to liabilities correlates strongly with lower risk. For the firms in the grey zone, most ratios exhibit low explanatory power, indicating that no single indicator has a strong impact.

Regarding the determinants of Investment Risk, the study concluded that past values of investment risk strongly predict future risk. Higher Political Stability and GDP growth decrease the risk of default. In contrast to GDP growth, GDP per capita indicates a non-linear relation with investment risk, which initially shows a negative effect, but after a certain threshold, it is found to decrease investment risk. Higher inflation is associated with lower risk, but it is found to have a smaller impact. Also, an improvement in the Current account balance is found to lower investment risk. Higher broad money, introduced in the model to capture the effects of monetary policy and financial condition in the economy, was found to increase the investment risk. This could be because when money becomes cheaper and more accessible, firms take on more debt to finance operations and growth, which eventually increases the risk of default. Among the internal factors, the Dividend premium significantly reduces the risk for investors, as it increases investor confidence in firms that send a strong signal about the firm’s financial health, stability, and commitment to reward investors. Accumulation of fixed assets is found to be associated with higher risk. Tax expenses show a negative but insignificant effect on risk. The explanatory variable firm size shows that larger firms face a higher risk of default. Finally, growth opportunities (book value per share) show a positive effect, pointing out that growth opportunities reduce default risk.

Overall, this comprehensive analysis contributes valuable insights for investors and policymakers in Pakistan. The study emphasises the need for a robust understanding of investment risk factors to facilitate informed decision-making.

## 6. Policy recommendations

On the basis of the findings, the study recommended the following policy measures.

- Firstly, investment risk is found to be sensitive to macro factors like political stability, GDP growth, inflation, and current account balance. Policies targeting macroeconomic conditions and promoting exports will definitely reduce investment uncertainty. In connection, it is desirable to improve current account resilience via export promotion and import substitution where feasible, focusing on tradable goods in growth clusters.
- Secondly, as the Textile sector is the dominant sector in the distress zone and also in the sample, the study recommends that diversification is needed to reduce sector-specific risk. Incentivising technology upgradation and process modernisation in the textile sector may raise productivity, which may lead to a decrease in the risk of default.
- Further results indicate that the market value of equity relative to liabilities lowers risk, dividend policies also appear to give a signal regarding good financial health, and large firms face higher risk. Hence, the study proposes to encourage dividend-linked financing instruments that channel profits into reinvestment.
- The study also recommends creating accessible corporate governance and financial management standards for firms to sustain investor confidence.
- Also, the government should promote transparent financial reporting programs to improve access to credit and reduce information asymmetries.

## 7. Limitations and future implications

The assessment is based on the publicly available data set of non-financial firms listed in the Pakistan Stock Exchange; hence, findings are constrained by data limitations, particularly, only the accounting variables are available for analysis within the dataset. The assessment hence relies on standard financial ratios, with key explanatory factors missing, such as the number of employees and their wage levels, actual production volumes, workforce composition by gender and roles, board demographics (including gender composition), firm age, ownership structure, and non-financial performance measures such as quality, innovation, and operational efficiency. The absence of the information has restricted the model from fully capturing organisational governance and operational drivers of risk. Furthermore, the model’s adaptability allows its application across various economic environments, making it a valuable tool for policymakers, financial institutions, and investment professionals. Hence, advancements in AI and machine learning can further refine this model, improving its predictive capabilities and expanding its usability for global investment markets.

## Supporting information

S1 Table Correlation between the financial variables and the Z-score for the Firms in the Distress Zone. (PDF)

S2 Table Correlation between the financial variables and the Z-score for the Firms in the Grey Zone. (PDF)

S3 Table Correlation between the financial variables and the Z-score for the Firms in the Safe Zone. (PDF)

S4 Table Variable definitions. (PDF)
