# Supplementary material for: Assessing the investment risk: An empirical analysis of Altman’s Z-score model
Source: PLoS One. 2026 Jul 30;21(7):e0354297. doi: 10.1371/journal.pone.0354297 (PMC13422875; doi:10.1371/journal.pone.0354297)
Supplement: S1 Table — (PDF) [file pone.0354297.s001.pdf]

S1 Table: Correlation between the financial variables and the Z-score for the Firms in the Distress Zone

Variable	*2014*		*2015*		*2016*		*2017*		*2018*		*2019*		*2020*		*2021*		*2022*		*2023*	
	Corr.	Rel.	Corr.	Rel.	Corr.	Rel.	Corr.	Rel.	Corr.	Rel.	Corr.	Rel.	Corr.	Rel.	Corr.	Rel.	Corr.	Rel.	Corr.	Rel.
WC/TA	-0.07	P	-0.13	P	0.17	P	-0.16	P	0.04	P	-0.16	P	-0.04	P	-0.06	P	-0.07	P	-0.03	P
RE/TA	0.99	S	0.89	S	0.81	S	0.97	S	0.94	S	0.90	S	0.98	S	0.99	S	0.99	S	0.99	S
EBIT/TA	-0.19	P	-0.01	P	0.07	P	-0.11	P	0.23	P	0.26	W	0.27	W	-0.90	S	-0.14	P	0.02	P
MVE/TL	0.76	S	0.66	M	0.23	P	0.66	M	0.70	M	0.69	M	0.54	M	0.06	P	0.50	W	0.55	M

Source: Authors 'Illustration. Here, "Corr" stands for correlations, and "Rel" indicates relationships. The relationships are categorised as P – Poor, S – Strong, W – Weak, and M – Moderate.
