# Supplementary material for: Assessing the investment risk: An empirical analysis of Altman’s Z-score model
Source: PLoS One. 2026 Jul 30;21(7):e0354297. doi: 10.1371/journal.pone.0354297 (PMC13422875; doi:10.1371/journal.pone.0354297)
Supplement: S2 Table — (PDF) [file pone.0354297.s002.pdf]

S2 Table: Correlation between the financial variables and the Z-score for the Firms in the Grey Zone

Variable	*2014*		*2015*		*2016*		*2017*		*2018*		*2019*		*2020*		*2021*		*2022*		*2023*	
	Corr.	Rel.	Corr.	Rel.	Corr.	Rel.	Corr.	Rel.	Corr.	Rel.	Corr.	Rel.	Corr.	Rel.	Corr.	Rel.	Corr.	Rel.	Corr.	Rel.
WC/TA	0.35	W	-0.06	P	-0.35	W	0.11	P	0.04	P	-0.47	W	-0.01	P	-0.31	W	-0.46	W	-0.07	P
RE/TA	0.45	W	0.44	W	0.61	M	0.13	P	0.55	M	0.45	W	0.42	W	0.40	W	0.67	M	0.43	W
EBIT/TA	0.14	P	0.27	P	-0.21	P	0.17	P	-0.53	M	0.42	W	-0.17	P	0.08	P	0.38	W	-0.33	W
MVE/TL	0.41	W	0.17	P	0.31	W	0.32	W	-0.17	P	-0.01	P	0.38	W	0.41	W	-0.27	W	0.53	M

Source: Authors 'Illustration. Here, "Corr" stands for correlations, and "Rel" indicates relationships. The relationships are categorised as P – Poor, S – Strong, W – Weak, and M – Moderate.
