# Supplementary material for: Assessing the investment risk: An empirical analysis of Altman’s Z-score model
Source: PLoS One. 2026 Jul 30;21(7):e0354297. doi: 10.1371/journal.pone.0354297 (PMC13422875; doi:10.1371/journal.pone.0354297)
Supplement: S3 Table — (PDF) [file pone.0354297.s003.pdf]

S3 Table: Correlation between the financial variables and the Z-score for the Firms in the Safe Zone

Variable	*2014*		*2015*		*2016*		*2017*		*2018*		*2019*		*2020*		*2021*		*2022*		*2023*	
	Corr.	Rel.	Corr.	Rel.	Corr.	Rel.	Corr.	Rel.	Corr.	Rel.	Corr.	Rel.	Corr.	Rel.	Corr.	Rel.	Corr.	Rel.	Corr.	Rel.
WC/TA	0.14	P	0.34	W	0.15	P	0.15	P	0.10	P	0.06	P	0.06	P	0.00	P	0.03	P	0.02	P
RE/TA	0.44	W	0.33	W	0.46	W	0.45	W	0.48	W	0.41	W	0.32	W	0.40	W	0.41	W	0.37	W
EBIT/TA	0.31	W	0.21	P	0.35	W	0.33	W	0.29	W	0.21	P	0.18	P	0.18	P	0.15	P	0.25	P
MVE/TL	0.93	S	0.95	S	0.90	S	0.89	S	0.90	S	0.92	S	0.92	S	0.93	S	0.92	S	0.89	S

Source: Authors 'Illustration. Here, "Corr" stands for correlations, and "Rel" indicates relationships. The relationships are categorised as P – Poor, S – Strong, W – Weak, and M – Moderate.
