# Supplementary material for: Assessing the investment risk: An empirical analysis of Altman’s Z-score model
Source: PLoS One. 2026 Jul 30;21(7):e0354297. doi: 10.1371/journal.pone.0354297 (PMC13422875; doi:10.1371/journal.pone.0354297)
Supplement: S4 Table — (PDF) [file pone.0354297.s004.pdf]

S4 Table: Variable Definitions

Variables	
**Liquidity**	Working Capital divided by Total Assets
**Leverage**	Retained Earnings divided by Total Assets
**Profitability**	Earnings before Interest and Tax payment divided by Total Assets
**Solvency**	Market Value of Equity divided by Total Liabilities
**GDP Growth**	GDP growth (annual %)
**Log GDP per Capita**	Log of GDP per capita (current US$)
**Political Stability**	Political Stability Index taken from Freedom in the World (Freedom House database). The index is based on the ratings of a 0–100 scale, the scores are derived from 25 indicators grouped into the political rights and civil liberties categories.
**Inflation Rate**	Inflation, consumer prices (annual %)
**Current Account Balance**	Current account balance (% of GDP)
**Broad Money**	Broad money growth (annual %)
**Dividend Payment**	Sum of Cash dividends and Stock dividends Paid
**Interest Payment**	Interest or markup payables
**Tax expenses**	Tax expenses Payables
**Operating Fixed Assets**	Operating fixed assets after deducting accumulated depreciation
**Growth opportunities**	Book value per share (Rs/share)
**Firm Size**	Measured through the natural logarithm of the total assets
